# Glycine is a transmitter in the human and chimpanzee cochlear nuclei

**DOI:** 10.3389/fnana.2024.1331230

**Published:** 2024-02-15

**Authors:** Joan S. Baizer, Chet C. Sherwood, Patrick R. Hof, James F. Baker, Sandra F. Witelson

**Affiliations:** ^1^Department of Physiology and Biophysics, Jacobs School of Medicine and Biomedical Sciences, University at Buffalo, Buffalo, NY, United States; ^2^Department of Anthropology, The George Washington University, Washington, DC, United States; ^3^Nash Family Department of Neuroscience and Friedman Brain Institute, Icahn School of Medicine at Mount Sinai, New York, NY, United States; ^4^Department of Neuroscience, Northwestern University Medical School, Chicago, IL, United States; ^5^Department of Psychiatry and Behavioural Neurosciences, Michael G. DeGroote School of Medicine, McMaster University, Hamilton, ON, Canada

**Keywords:** inhibition, immunohistochemistry, brainstem, audition, tinnitus, hyperacusis

## Abstract

**Introduction:**

Auditory information is relayed from the cochlea via the eighth cranial nerve to the dorsal and ventral cochlear nuclei (DCN, VCN). The organization, neurochemistry and circuitry of the cochlear nuclei (CN) have been studied in many species. It is well-established that glycine is an inhibitory transmitter in the CN of rodents and cats, with glycinergic cells in the DCN and VCN. There are, however, major differences in the laminar and cellular organization of the DCN between humans (and other primates) and rodents and cats. We therefore asked whether there might also be differences in glycinergic neurotransmission in the CN.

**Methods:**

We studied brainstem sections from humans, chimpanzees, and cats. We used antibodies to glycine receptors (GLYR) to identify neurons receiving glycinergic input, and antibodies to the neuronal glycine transporter (GLYT2) to immunolabel glycinergic axons and terminals. We also examined archival sections immunostained for calretinin (CR) and nonphosphorylated neurofilament protein (NPNFP) to try to locate the octopus cell area (OCA), a region in the VCN that rodents has minimal glycinergic input.

**Results:**

In humans and chimpanzees we found widespread immunolabel for glycine receptors in DCN and in the posterior (PVCN) and anterior (AVCN) divisions of the VCN. We found a parallel distribution of GLYT2-immunolabeled fibers and puncta. The data also suggest that, as in rodents, a region containing octopus cells in cats, humans and chimpanzees has little glycinergic input.

**Discussion:**

Our results show that glycine is a major transmitter in the human and chimpanzee CN, despite the species differences in DCN organization. The sources of the glycinergic input to the CN in humans and chimpanzees are not known.

## 1 Introduction

Auditory information is relayed from the cochlea via the eighth cranial nerve to the cochlear nuclei (CN). Based on anatomical studies in cats and rodents, the CN are divided into four subdivisions: the dorsal cochlear nucleus (DCN), the granule cell domain (Gr), and two subdivisions of the ventral cochlear nucleus (VCN), the posterior (PVCN) and anterior (AVCN) nuclei (Harrison and Irving, 1965, 1966; Osen, 1969; Brawer et al., 1974; Hackney et al., 1990; Morest et al., 1990; Cant, 1992; Trettel and Morest, 2001; Mylius et al., 2013). Anatomical and electrophysiological studies have identified classes of neurons with distinct spatial distributions in the CN. The DCN includes fusiform cells, cartwheel cells, granule cells, and tuberculoventral cells (reviewed in Wouterlood and Mugnaini, 1984; Oertel and Young, 2004). Octopus cells are found in a region of the caudal PVCN, the octopus cell area (OCA), and spherical bushy cells in a region in the rostral AVCN (Harrison and Irving, 1966; Osen, 1969; Bacsik and Strominger, 1973; Brawer et al., 1974; Mugnaini et al., 1980; Hackney et al., 1990; Morest et al., 1990; Cant, 1992; Mylius et al., 2013). Globular bushy cells are found more caudally in the AVCN near the auditory nerve root (Tolbert and Morest, 1982; Spirou et al., 1990; Rhode, 2008). Multipolar/stellate cells are found in both PVCN and AVCN (Cant, 1981; Doucet et al., 1999; Ngodup et al., 2020).

Several lines of evidence have established glycine as a major inhibitory transmitter in the CN of rodents and cats. Neurons expressing glycine receptors have been described in the DCN and VCN (Altschuler et al., 1986, rat, guinea pig; Sanes et al., 1987, gerbil; Aoki et al., 1988, rat; Araki et al., 1988, rat; Glendenning and Baker, 1988, cat; Lin and Xie, 2019, mouse). Glycinergic axons and terminals have been found in the CN of rats (except in the OCA; Zafra et al., 1995b; Friauf et al., 1999). One source of glycinergic input is from the glycinergic CN cells: the cartwheel cells and tuberculoventral cells of the DCN, and the commissural, D-stellate and L-stellate cells of the VCN are all glycinergic (Wenthold et al., 1987; Oertel et al., 1990; Saint Marie et al., 1991; Gates et al., 1996; Golding and Oertel, 1996; Alibardi, 1999a,b; Ngodup et al., 2020). Another possible source is projections from glycinergic neurons in the medial nucleus of the trapezoid body (MNTB), a component of the superior olivary complex (SOC; Aoki et al., 1988; Helfert et al., 1989).

Anatomical experiments have found differences in the organization of the CN in primates compared to rodents and cats. Three of the four major divisions (DCN, AVCN, and PVCN) are found (Bacsik and Strominger, 1973; Heiman-Patterson and Strominger, 1985; Baizer et al., 2014, 2018a). However, the granule cell domain is decreased or absent in primates (Moore and Osen, 1979; Moore, 1980, 1987; Heiman-Patterson and Strominger, 1985; Adams, 1986; Moore et al., 1996). The laminar organization of the DCN is much less well defined in humans (Moore and Osen, 1979; Heiman-Patterson and Strominger, 1985; Adams, 1986; Baizer et al., 2014). Fusiform neurons in the DCN of primates lack the orderly arrangement and orientation seen in other species (Moskowitz, 1969; Moore and Osen, 1979; Heiman-Patterson and Strominger, 1985; Moore, 1987; Baizer et al., 2014). Adams (1986) thought that cartwheel, granule, and fusiform cells were all absent in the human DCN but did not mention tuberculoventral cells. Octopus cells, bushy cells, and stellate/multipolar cells have been found in the human VCN (Adams, 1986; Kulesza Jr, 2014). There are also species-related differences in the organization of the nuclei of the SOC (review in Alhelo et al., 2023). The medial nucleus of the trapezoid body (MNTB) is not consistently found in humans (Moore and Moore, 1971; Kulesza Jr and Grothe, 2015), and the lateral superior olive (LSO) is much less defined in humans than in rodents or cats (Moore and Moore, 1971).

These differences in DCN organization between primates and other species raise the possibility of differences in circuitry in the CN of primates. Glycine receptors have been described in many structures in the human brainstem (Baer et al., 2003, 2009), but those studies did not specifically mention the CN. Understanding the substrate of inhibition in the human CN is important since changes in inhibition have been implicated in human hearing disorders (Brozoski et al., 2002; Wang et al., 2009; Kaltenbach, 2011; Auerbach et al., 2014).

In the current study, we have investigated whether neurons in the human CN receive glycinergic input. This question is relatively easy to answer experimentally; immunoreactivity with antibodies to glycine receptors should mark neurons receiving glycinergic input as in other species (Altschuler et al., 1986, guinea pig; Araki et al., 1988, rat; Wenthold et al., 1988, guinea pig). Glycinergic input can also be examined by using an antibody to the neuronal glycine transporter (GLYT2) to label axons and terminals (Poyatos et al., 1997; Friauf et al., 1999; Uusisaari and Knopfel, 2010).

We have also considered potential species differences in glycinergic input to the CN. We compared the patterns of immunoreactivity with antibodies to glycine receptors and to the neuronal glycine transporter in humans with those in chimpanzees, a closely related primate. We also compared data in humans and chimpanzees with the cat to see if the results with our antibodies were consistent with earlier data on the distribution of glycinergic receptors in that species (Glendenning and Baker, 1988).

## 2 Materials and methods

### 2.1 Human brainstems

We studied human brainstems from the Witelson Normal Brain Collection; the details of subject selection and tissue acquisition were described in Witelson and McCulloch (1991). Briefly, the subjects were patients diagnosed with metastatic cancer. At the time of enrollment in the study, they had no diagnosed neurological disease. They then underwent neuropsychological testing focused on cognitive tasks and experimental dichotic listening tasks. Standard audiological testing was done for each subject, and each had hearing levels within normal limits for each ear and did not wear hearing aids. Patients were then followed medically and periodically screened for the development of neurologic dysfunction. At death, brains were removed by pathologists and examined for neuropathology. We selected cases with short post-mortem intervals (PMI) and no documented neurological dysfunction in life or neuropathology after death.

Table 1 shows the case number, age, sex, and PMI (in hours) of the human brain specimens we used. We have previously reported data on protein expression in various brainstem nuclei of these cases, including the CN (Baizer et al., 2007, 2011a,b, 2013b, 2014; Baizer and Broussard, 2010).

**Table 1:** Human cases (Witelson Normal Brain Collection).

Case	Age	Sex	PMI (h)
155	50	F	9
158	51	M	1
164	45	F	3
166	65	F	3
168	69	M	3
169	70	M	2
176	71	F	3
180	54	M	2

### 2.2 Histological procedures: human tissue

Our methods for processing this tissue have been described previously (Baizer et al., 2007, 2013b; Baizer and Broussard, 2010). Briefly, tissue blocks containing the brainstem and cerebellum were dissected away from the cerebrum, and all tissue was stored in 10% formalin. We further separated the brainstem and cerebellum and then cryoprotected the brainstems with 15% sucrose and 30% sucrose in 10% formalin. Prior to sectioning, we made a small slit along one side of the ventral brainstem to allow identification of the left and right sides of the brain. About 40-μm-thick frozen sections were cut on an American Optical (AO) sliding microtome in a plane transverse to the brainstem. All sections were collected and stored in plastic compartment boxes, five sections/compartments, at 4°C in 5% formalin. Initially, sets of sections 2 mm apart from each case were stained with a Nissl stain, cresyl violet, following a standard protocol (LaBossiere and Glickstein, 1976). Additional sections were stained as needed in different studies to define the limits of structures of interest.

### 2.3 Chimpanzee brainstems

We also processed archival sections from three chimpanzees (*Pan troglodytes*; cases AN, f, age 15; MT, m, age 25; and WM, f, age 13). Chimpanzees had been housed at the Emory National Primate Center according to Association for the Assessment and Accreditation of Laboratory Animal Care International (AAALAC) guidelines. All died from natural causes. None of the animals showed evidence of neurological disorder and appeared normal on routine assessments for pathology. Details of the histology for these cases have been published in earlier reports (Baizer et al., 2011b, 2013a, 2018b). Blocks of tissue, including the cerebellum and brainstem, were first cryoprotected in 15% and then 30% sucrose in phosphate-buffered saline (PBS), and then frozen, transverse sections of the brainstems were cut with an AO sliding microtome at 40 μm. Every section was collected and stored in a cryoprotection solution of 30% ethylene glycol, 25% glycerol, and 45% 0.1 M phosphate buffer at −20°C.

### 2.4 Cat brainstem

We immunostained archival sections of a cat brainstem that had been prepared for earlier studies (Baizer and Baker, 2005, 2006; Baizer et al., 2010). For those studies, 10 adult cats of both sexes were obtained from commercial breeders, maintained in the Northwestern University animal care facility, and handled according to the guidelines of the Northwestern University Animal Care and Use Committee. Frontal sections 50 μm thick were cut on the AO sliding microtome and stored in tissue culture wells in the cryoprotection solution in a −20°C freezer. We selected sections containing the CN and/or SOC and used the same antibodies and immunohistochemistry (IHC) protocols as in human and chimpanzee tissue, except for omitting the antigen retrieval protocol.

### 2.5 Archival Nissl and immunostained slides

We also examined sections from cats, chimpanzees, and humans that had been stained for cresyl violet or immunostained for non-phosphorylated neurofilament protein (NPNFP) or calretinin (CR) in the course of earlier studies (Baizer and Baker, 2005; Baizer and Broussard, 2010; Baizer et al., 2013a). NPNFP is a structural protein expressed in subsets of pyramidal cells and their axons in the cerebral cortex and in subsets of projection neurons in the brainstem (Hof et al., 1995; Hof and Morrison, 1995; Baizer et al., 2011a, 2013a). CR is a calcium-binding protein expressed by subsets of interneurons in the cerebral cortex (Glezer et al., 1992; Condé et al., 1994). In the auditory brainstem, neurons in the AVCN and octopus cells in the PVCN express CR (Lohmann and Friauf, 1996; Felix et al., 2017; Baizer et al., 2018a). Some of these sections had been immunostained with a DAB visualization protocol, giving brown staining in contrast to the gray/black staining seen with the glucose oxidase visualization protocol.

### 2.6 Antibodies and immunohistochemistry

All IHC was performed on free-floating sections. Sections were rinsed in PBS (all rinses were 3 × 10 min). Sections from humans and chimpanzees were then treated with an antigen retrieval (AR) protocol. Each section was placed in a separate small glass jar with 20 mL of citrate buffer (pH 6). The jars were heated in a water bath at 85°C for 30 min. The jars were removed from the bath and cooled to room temperature. Sections were rinsed in PBS, and non-specific labels were blocked by incubating sections in a solution of PBS, 1% Triton X-100, 1% bovine serum albumin, and 1.5% normal serum (from the appropriate Vector Elite kit). The primary antibody was added, and sections were incubated overnight at 4°C on a tissue rocker. Further processing was with the Vector “ABC” method using the appropriate Vector Elite kit (mouse or rabbit; Vector Laboratories, Burlingame, CA), followed by visualization with a 3,3′-diaminobenzidine (Sigma-Aldrich now Thermo Fisher Scientific) protocol, giving brown staining, or a glucose oxidase modification of the protocol, giving gray-black staining (Shu et al., 1988; Van der Gucht et al., 2006). Sections were mounted on gelled slides, dehydrated in 70, 95, and 100% alcohol, cleared in xylene, and coverslipped with Permount (Fisher Scientific). Table 2 shows the primary antibodies and dilutions used. The figure legends indicate which antibody was used for the sections shown.

**Table 2:** Antibodies and dilutions.

Antigen	Source, catalogue #	Host	Type	Dilution
CR	Chemicon, AB 5054	Rb	Polyclonal	1:1000–1:3000
GLYR	Abcam, ab23809	Rb	Polyclonal	1:1000
GLYR	Invitrogen/Millipore, Ab5052	Rb	Polyclonal	1:500
GLYR	Synaptic Systems, 146–111	Ms	Monoclonal	1:500
GLYT2	Alomone Laboratories, AGT-012	Rb	Polyclonal	1:100–1:200
GLYT2	Santa Cruz, sc-390090	Ms	Monoclonal	1:200
NPNFP	Covance, SMI32	Ms	Monoclonal	1:1000

### 2.7 Antibody specificity and controls

#### 2.7.1 Glycine receptors and transporters

The glycine receptor is composed of alpha (α) and beta (β) subunits, with several α isoforms identified (Rajendra et al., 1997; Lynch, 2009). The structure of the α subunits is similar across humans, rats, and mice (references in Rajendra et al., 1997). We used antibodies to the α1 or α1 and α2 subunits.

There are two glycine transporters, GLYT1 and GLYT2; GLYT1 is expressed in glial cells, and GLYT2 is expressed in neurons and axon terminals (Zafra et al., 1995a). We used antibodies to GLYT2.

##### 2.7.1.1 Glycine receptor antibodies

Abcam, ab23809. RRID:AB_2110062 – Rabbit polyclonal to glycine receptor subunits α1 + α2. The immunogen was a rat synthetic peptide. The antibody recognizes an epitope within the N-terminal region of glycine receptor α1 and α2 subunits. The positive controls were rat brain lysate and rat spinal cord lysate. By Western blot, it recognized a band of about 48 kDa (according to the manufacturer). It has been used in a study of the auditory brainstem in mice (Yoo et al., 2015) and in the retina of rats (Li et al., 2020).

Millipore/chemicon, Ab5052. RRID:AB_91659 – Rabbit anti-glycine receptor, affinity purified. It recognizes the glycine receptor subunit α1 with cross-reactivity with the α2 subunits. The immunogen is a peptide from the N terminus of the human glycine receptor (sequence ARSTKPMSPSDFLDKLMGC). By Western blot, it recognized a band of about 48 kDa, according to the manufacturer. It has been used in studies of rats and humans (Geiman et al., 2002; Rubio and Juiz, 2004; Baer et al., 2009).

Synaptic systems, 146–111. RRID:AB_887723 – Mouse monoclonal antibody that recognizes epitopes AA29–AA39 from rat glycine receptor α1. The immunogen is a recombinant protein corresponding to AA1–457 from rat glycine receptor α1, according to the manufacturer. It has been used in studies of mice, rats, and humans (Hruskova et al., 2012; Langlhofer et al., 2020; Rauschenberger et al., 2020).

##### 2.7.1.2 Neuronal glycine transporter antibodies

Alomone Laboratories, AGT-012. RRID:AB_11121049 – Rabbit polyclonal antibody to the neuronal glycine transporter, GLYT2. The immunogen is peptide CVIGDHPKIQIKNS, corresponding to amino acid residues 333–346 of the rat glycine transporter’s second extracellular loop. In Western blots, it recognized a band at about 100 kDa (mouse brain lysates and rat cerebellum, according to the manufacturer). It has been used in the avian spinal cord (Stanchak et al., 2022).

Invitrogen/Millipore, PA5-69264. RRID: AB_2689492 – Rabbit polyclonal antibody; the immunogen was a synthetic peptide directed against the N-terminal region of human SLC6A5. It was purified by affinity chromatography. Western blot data were not available from the manufacturer; however, the immunostaining patterns with this antibody were comparable to those of the other GLYT2 antibodies we used.

Santa Cruz, sc-3090090. RRID is not available – Mouse monoclonal antibody raised against amino acids 50–204 mapping near the N terminus of human GLYT2. The positive control was rat brain extract (sc2392). On a Western blot of brain tissue extract, there was a dark band at about 105 kDa and a lighter band at about 76 kDa, according to the manufacturer.

##### 2.7.1.3 Control sections for immunohistochemistry

In order to control for non-specific myelin labels with secondary antibodies (Weruaga et al., 1998), we processed control sections from each species (human, chimpanzee, and cat) according to the standard protocols (antigen retrieval protocol used for human and chimpanzee sections but not cat) with both vector kits (rabbit and mouse) omitting the primary antibody. No immunostaining was seen on the control sections.

##### 2.7.1.4 Antibodies for archival slides

Calretinin (CR). Chemicon/Millipore, #AB5054. RRID:AB_2068506 – Rabbit polyclonal antibody to recombinant rat calretinin. The calretinin antiserum recognized a band of 31 kDa on the Western blot of the rat brain (according to the manufacturer). No immunostaining was seen in sections in which the antiserum was diluted (1, 1,500 in 1.5 cc of antibody diluent) and preincubated with the CR protein (Swant, recombinant human CR produced in *E. coli*, 3 μg/mL) for 6 h at 4°C prior to immunostaining (following Swant instructions).

Non-phosphorylated neurofilament protein (NPNFP). Covance #SMI-32. RRID:AB_2315331 – This is a mouse monoclonal antibody to non-phosphorylated neurofilament protein (NPNFP). This antibody recognizes a non-phosphorylated epitope on the 168 (M, medium) and 200 kDa (H, heavy) neurofilament subunits (Sternberger and Sternberger, 1983) of most mammalian species and stains somata, dendrites, and some thick axons (manufacturer’s data). It lacks cross-reactivity to microtubule-associated protein and to Alzheimer’s disease neurofibrillary tangles (Ksiezak-Reding et al., 1987; Lee et al., 1988).

### 2.8 Data analysis and photography

We examined sections with a Leitz Dialux 20 light microscope, using objectives 1.6× (numerical aperture 0.05), 2.5× (0.08), 2.5× (0.08), 6.3× (0.22), 10× (0.30), and 25× (0.65). We captured digital images (1,600 × 1,200 pixels) with a SPOT Insight Color Mosaic camera. Brightness, contrast, and color of the images were adjusted, and figures were assembled with Adobe Photoshop software (San Jose, CA).

## 3 Results

### 3.1 Humans: distribution of the glycine receptor

**Figure 1:**
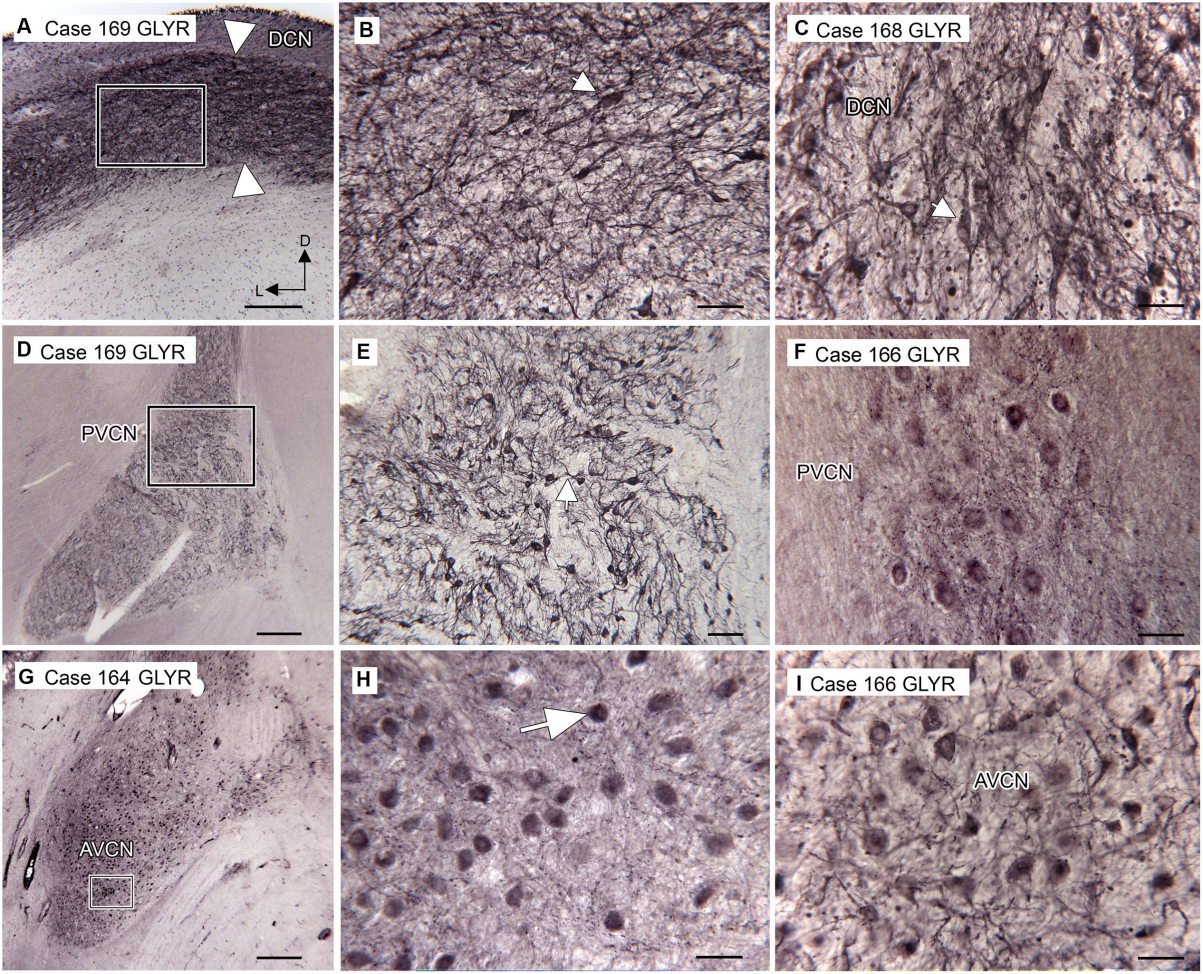
Immunoreactivity to glycine receptors (GLYR) in the human CN. **(A)** Case 169, DCN. There is a dense band of label (between the arrowheads) about 200 μm below the surface of the brainstem and running parallel to it. The rectangle shows the location of the higher magnification image in **B** (Abcam antibody). **(B)** The higher magnification image shows that the immunolabel consists of labeled somata (example at arrow) embedded in a meshwork of stained fibers running at all orientations. **(C)** Immunoreactivity to GLYR in the DCN of Case 168, showing immunolabeled somata (example at arrow) and processes (Abcam antibody). **(D)** Case 169. Low magnification image showing the PVCN, which is found ventral to the DCN at this level (Abcam antibody). The rectangle shows the location of the image in **E**, in which many labeled somata and proximal processes (example at arrow) can be seen. **(F)** Immunolabeled somata in the PVCN of Case 166 (Synaptic Systems antibody). **(G)** Immunolabel in the AVCN of Case 164; there is a prominent immunolabel of somata throughout the AVCN. The rectangle shows the location of the higher magnification image to the right (Synaptic Systems antibody). **(H)** Immunoreactive round somata (example at larger arrow) and puncta. **(I)** Immunolabeled somata in the AVCN of Case 166 (Invitrogen antibody). Scale bars **D,G** = 500 μm; **A** = 250 μm; **E** = 100 μm; **B,C,F,H,I** = 50 μm. The orientation bars in A indicate dorsal (D) and lateral (L) and apply to all panels.

**Figure 2:**
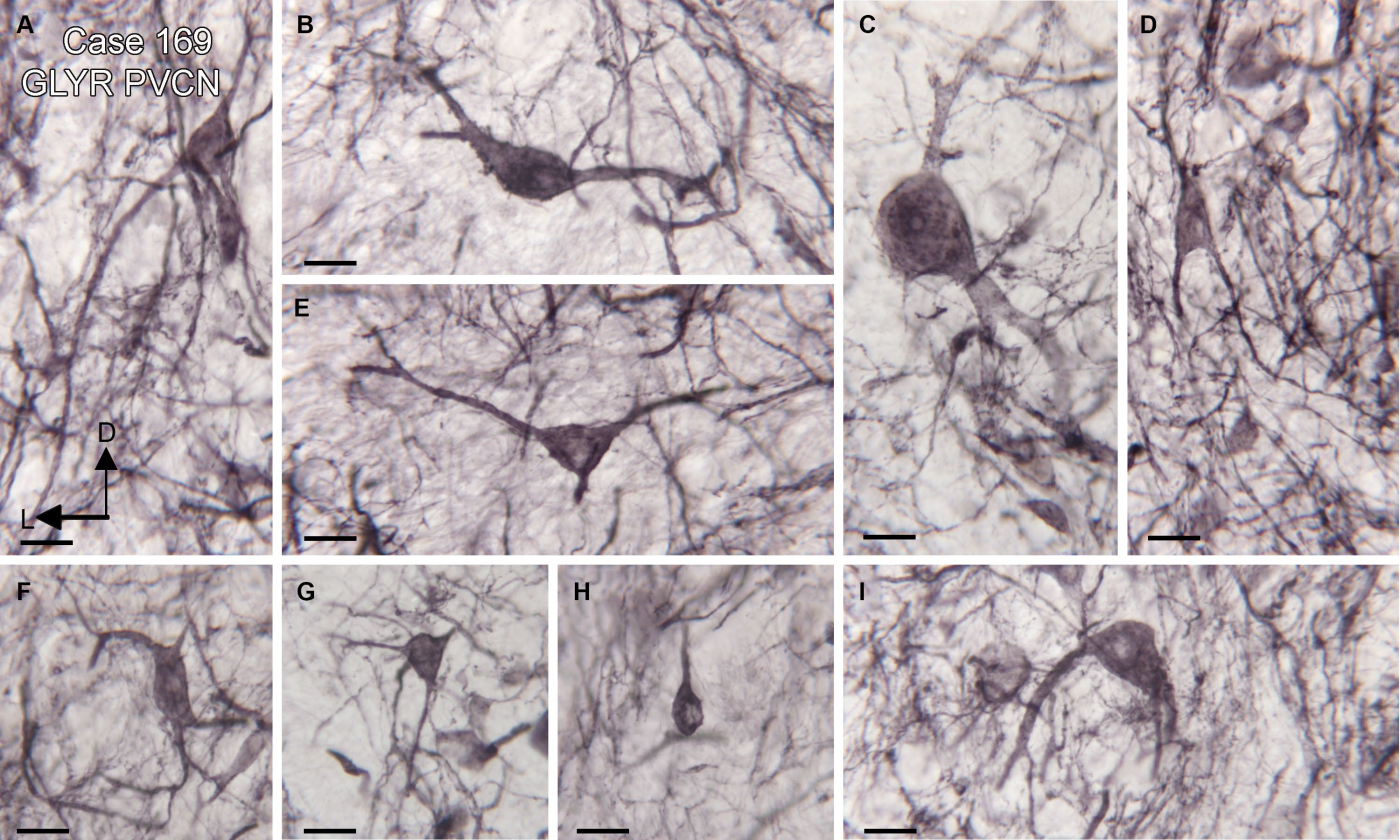
Diversity of GLYR-ir neurons in the PVCN, Case 169, the section is shown in Figures 1D,E. **(A–I)** Higher magnification images of individual neurons in the PVCN. Scale bars for all panels = 20 μm. The orientation bars in A indicate dorsal (D) and lateral (L) and apply to all panels.

We found expression of glycine receptors on somata and processes of neurons in all CN subdivisions, the DCN, PVCN, and AVCN, in each case examined. Figure 1A shows the immunolabel in the DCN. There is a band of label (between the white arrowheads) about 200 μm below the surface of the DCN and about 400 μm wide, running parallel to the surface. This band is composed of immunolabeled somata (example in Figure 1B, arrow) embedded in a meshwork of immunostained processes running at all orientations. Figure 1C shows immunolabeled neurons in the DCN of a second case. Figures 1D,E show the PVCN on this section. There are many immunolabeled somata of a variety of shapes; the proximal dendrites of many neurons are labeled (example in Figure 1E, arrow). There is a range of soma sizes, shapes, and orientations of both somata and dendrites relative to the dorsoventral axis of the PVCN. Figure 1F shows immunolabeled somata and puncta in the PVCN of another case. Figures 1G,H show immunostaining in the AVCN. There are immunostained neurons with mainly round somata (example in Figure 1H, arrow) throughout, as well as scattered puncta. A similar staining pattern is shown for another case in Figure 1I. The diversity of GLYR-ir neuron types in the PVCN is also illustrated in Figure 2, which shows higher magnification images of nine GLYR-ir neurons from the section shown in Figures 1D,E.

**Figure 3:**
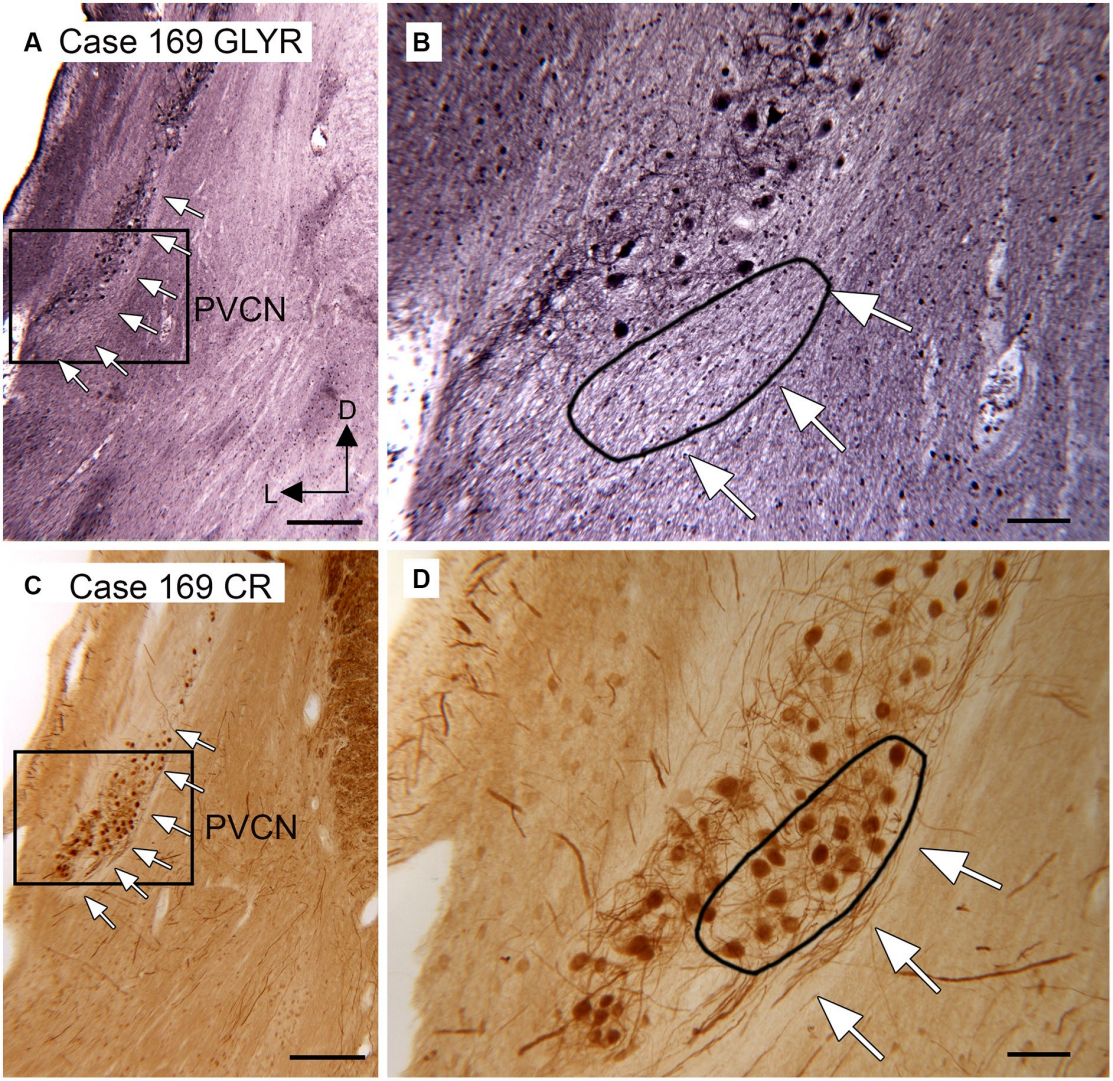
Paucity of neurons showing immunoreactivity to glycine receptors in the putative location of the human OCA. **(A)** Section through the DCN and PVCN of Case 169 showing immunoreactivity to the glycine receptor (Abcam antibody). The white arrows show the medial border of the PVCN. The rectangle shows the region of the higher magnification image in **B**. **(B)** The white arrows show the medial border of the PVCN, and the black outline shows a region in which there are no GLYR-ir neurons. **(C)** Section about 200 μm caudal to the one in A immunostained for CR. The white arrows show the medial border of the PVCN; the rectangle shows the region shown at higher magnification in **D**. **(D)** The white arrows show the medial border of the PVCN, and the black outline shows a region with a high density of darkly labeled CR-ir neurons. Scale bars: **A,C** = 500 μm; **B,C** = 100 μm; inset = 20 μm. The orientation bars in **A** indicate dorsal (D) and lateral (L) and apply to all panels.

Anatomical and physiological studies in mice and rats have suggested that octopus cells in those species receive little, if any, glycinergic input (Golding et al., 1995; Zafra et al., 1995a; Friauf et al., 1999). We asked whether that might also be true in humans and chimpanzees. Octopus cells in several species have been shown to express the calcium-binding protein calretinin (CR; Lohmann and Friauf, 1996; Adams, 1997; Kulesza Jr, 2014; Felix et al., 2017). We compared the distribution of glycine receptor (GLYR)-immunoreactive (ir) cells in the PVCN with the distribution of CR-ir neurons. Figure 3 shows immunostaining with the two antibodies on two sections about 200 μm apart. Immunoreactivity to GLYR (Figures 3A,B) shows a ventral and lateral cluster of immunolabeled cells in the PVCN. The white arrows (Figure 3A) show the medial border of the PVCN. The higher magnification image (Figure 3B) shows GLYR-ir neurons more laterally in the PVCN. The black line encloses a medial region in which no immunolabeled neurons are found. The distribution of these neurons is compared with the distribution of CR-ir neurons on a section about 200 mm caudal (Figures 3C,D). The white arrows in Figure 3C show the lateral border of PVCN with CR-ir neurons found relatively medially. The higher magnification image shows that there is a cluster of CR-ir neurons within the region in which there were no GLYR-ir neurons (black outline). This result is consistent with a lack of glycinergic input to at least part of the OCA in humans.

### 3.2 Human: distribution of glycinergic axons and terminals

**Figure 4:**
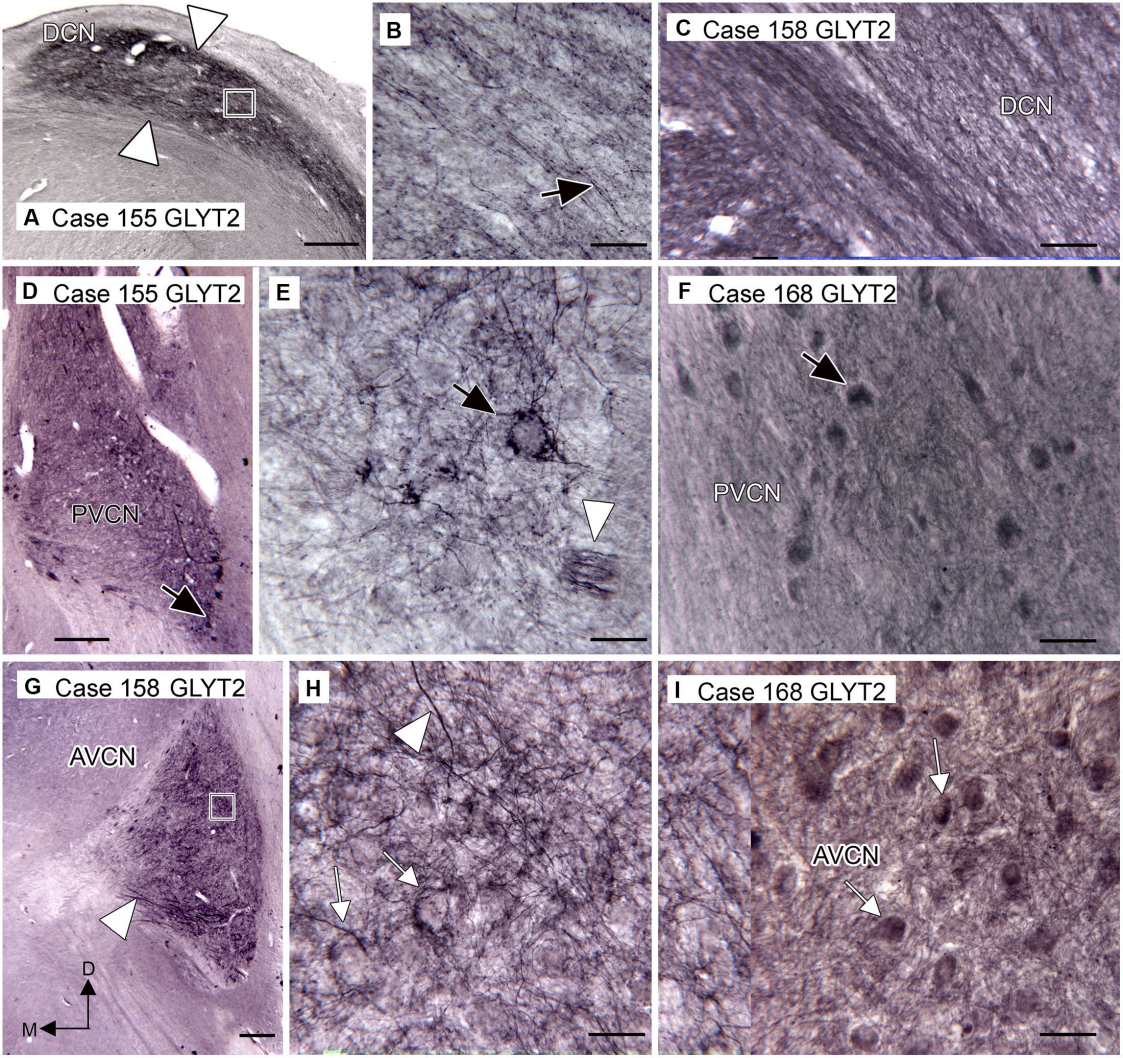
Immunoreactivity to GLYT2 in the human CN (Santa Cruz antibody, **A,B,D,E,G,H**). **(A)** Case 155, DCN. There is a band of label, indicated by white arrowheads, below the surface of the brainstem and running parallel to it. The rectangle shows the location of the image to the right. **(B)** The higher magnification image shows that the immunolabel is composed of many fibers (example at arrow), some beaded, running parallel to the band, as well as numerous scattered puncta. **(C)** Immunolabel in the DCN in Case 158 showing fibers and puncta (Invitrogen antibody). **(D)** Case 155, PVCN. At lower magnification, there is label ventromedially. The black arrow shows the location of the neuron illustrated in **E**. **(E)** The higher magnification image shows immunolabeled processes surrounding this neuron (black arrow). The white arrowhead shows a small fascicle of immunolabeled fibers. **(F)** Immunolabeled neurons in the PVCN of Case 168 (Invitrogen antibody). **(G)** Immunostaining in the AVCN of Case 158. The lower magnification image shows that there is patchy labeling throughout the AVCN and a band of immunolabeled fibers ventrally (arrowhead). The rectangle shows the location of the higher magnification image to the right. **(H)** The higher magnification image shows immunolabeled fibers running at all angles (example at arrowhead) and puncta. The arrows show examples of somata that are surrounded by labeled processes. **(I)** GLYT2-ir in the AVCN of Case 168 shows immunolabeled neurons (examples at arrows) and processes (Invitrogen antibody). Scale bars: **A,D,G** = 500 μm; **B,C,E,F,H,I** = 50 μm. The orientation bars in **G** indicate dorsal (D) and medial (M) and apply to all panels.

The distribution of glycinergic input to the CN, as shown by immunoreactivity to the neuronal glycine transporter (GLYT2), aligns well with the distribution of neurons expressing the glycine receptor. We saw immunolabeled fibers and puncta in the DCN (Figures 4A–C), PVCN (Figures 4D–F), and AVCN (Figures 4G–I). Figure 4A shows that immunolabeling for GLYT2 identifies a band of label in the DCN that is similar in width and position to the band shown in Figure 3A. This band consists of labeled processes, many running parallel (Figure 4B, arrow; Figure 4C), and many scattered labeled puncta. Figure 4D shows immunolabel medially in VCP on the same section. The arrow indicates a neuron surrounded by labeled fibers, as shown at higher magnification in Figure 4E. The arrowhead in Figure 4E shows a small band of immunolabeled fibers Immunolabeled neurons are also shown in Case 168 in Figure 4F (example at arrow). More rostrally, in the AVCN (Figures 4G–I), there are somata encircled by immunolabel (Figures 4H,I examples at arrows) as well as labeled fibers throughout (example at arrowhead in Figure 4H).

### 3.3 Chimpanzee

**Figure 5:**
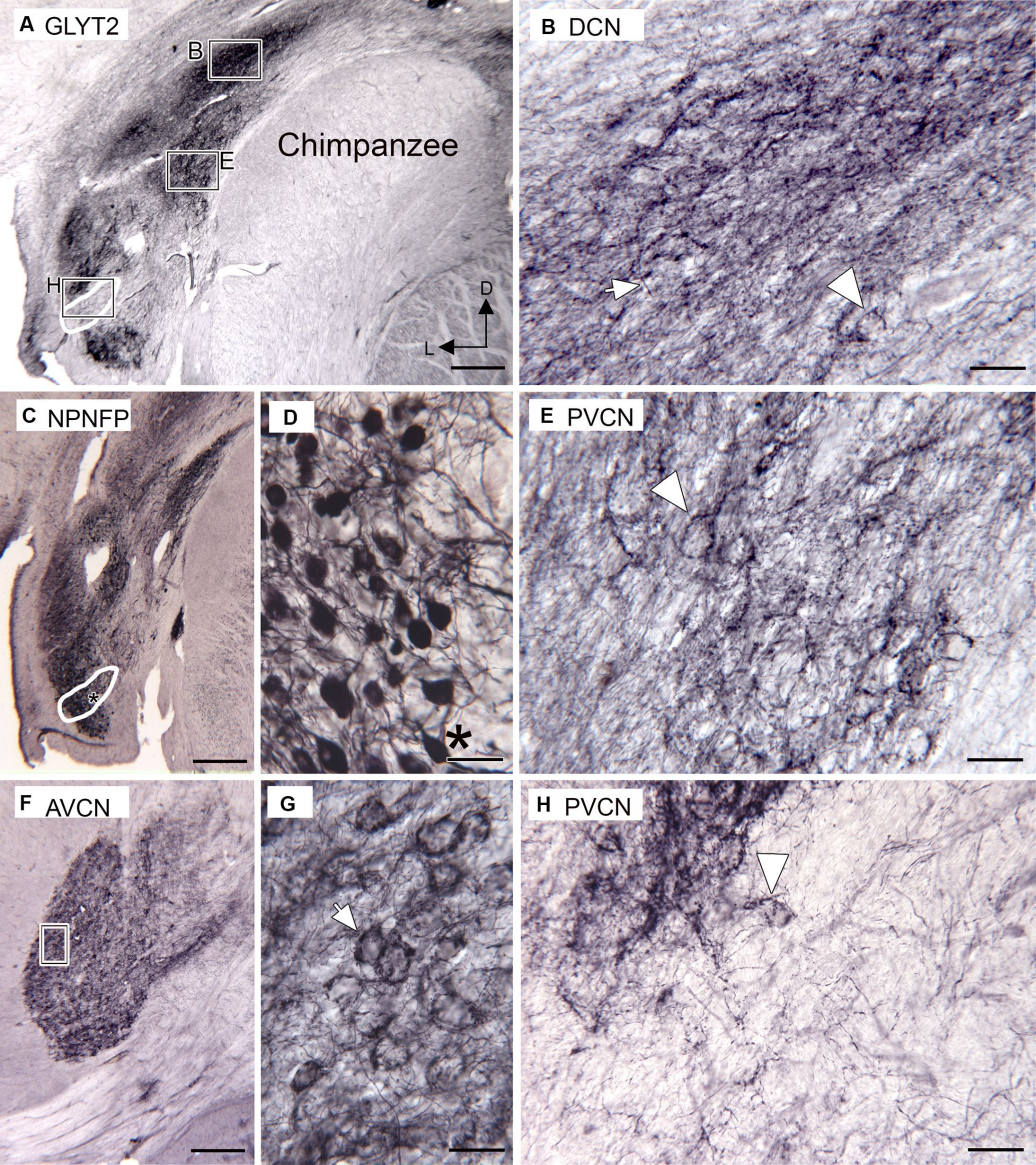
Immunoreactivity to the glycine transporter in chimpanzee CN (Santa Cruz antibody). **(A)** Immunoreactivity in the DCN and PVCN. There is a dense band of immunoreactivity in the DCN. The rectangles show the location of the higher magnification images in **B**, **E**, **H**. The white outline shows an area with little immunostaining, in comparison with the section in **C**. **(B)** Higher magnification image of the DCN immunostaining. The arrow indicates a fiber, and the arrowhead is an outlined soma. **(C)** NPNFP immunoreactivity in the PVCN on a section about 1 mm caudal to the section shown in **A**. The white outline shows the location of the region with light GLYT-ir in **A**. The asterisk is an alignment point for the image in **D** showing higher magnification images of neurons. **(D)** NPNFP-ir neurons in PVCN within the region in which there was little immunoreactivity to GLYT2. The asterisk is an alignment point for **C** and shows a neuron resembling an octopus cell. **(F)** GLYT2 immunostaining in the AVCN; the section is about 2 mm rostral to the one in **A**. The rectangle marks the location of the image in **G**, which shows many round or oval somata outlined by immunostaining (example at arrow). **(H)** Higher magnification image of the region with very little GLY2-ir. The arrowhead indicates a cell dorsolateral to this area that is outlined by GLYT2 immunoreactivity. Scale bars: **A,C,F** = 500 μm; **B,E,G,H** = 50 μm; **E** = 100 μm.

We used Figures 4–6 in Strominger et al. (1977) and Figure 1 in Heiman-Patterson and Strominger (1985) as well as our own Nissl-stained sections, to identify the CN subdivisions in chimpanzees. Immunoreactivity to the neuronal glycine transporter suggests glycinergic input to DCN, PVCN, and AVCN. Figure 5A shows immunolabel in the DCN dorsally and the PVCN ventrally; the white outline shows a region of sparse immunolabel in the PVCN. The rectangles show the locations of the higher magnification images of the DCN (B), PVCN (E), and the region of light immunostaining (H). Figure 5B shows immunolabeled fibers (example at arrow) and puncta consistent with glycinergic axons in the DCN. Immunolabel also surrounds some somata (the arrowhead shows an example in deep DCN). Figure 5E shows the immunolabel in the PVCN. There are immunolabeled fibers and puncta, and many neurons are also surrounded by immunolabels (example at arrowhead). Figure 5H shows a higher magnification image of the region with a light GLYT2 immunolabel. The arrowhead shows a neuron at the dorsolateral edge of this region that is surrounded by immunolabel. Only a few immunolabeled fibers cross the rest of that region. Figure 5C shows a section about 1 mm caudal immunostained for NPNFP. The white outline shows the location of the region defined in A with a very light immunolabel. There are NPNFP-ir neurons, some resembling octopus cells (Figures 5C,D, neuron at asterisk) in the region outlined in white. These images are consistent with the suggestion that the OCA in the chimpanzee may lack glycinergic input. In the AVCN (Figures 5F,G), there is also immunolabeling surrounding many neurons. The lower magnification image shows that the immunoreactivity in the AVCN is distributed throughout. The higher magnification image (Figure 5G) shows immunolabel surrounding round or oval somata; these are likely bushy cells (example at arrow in Figure 5G).

### 3.4 Cat

**Figure 6:**
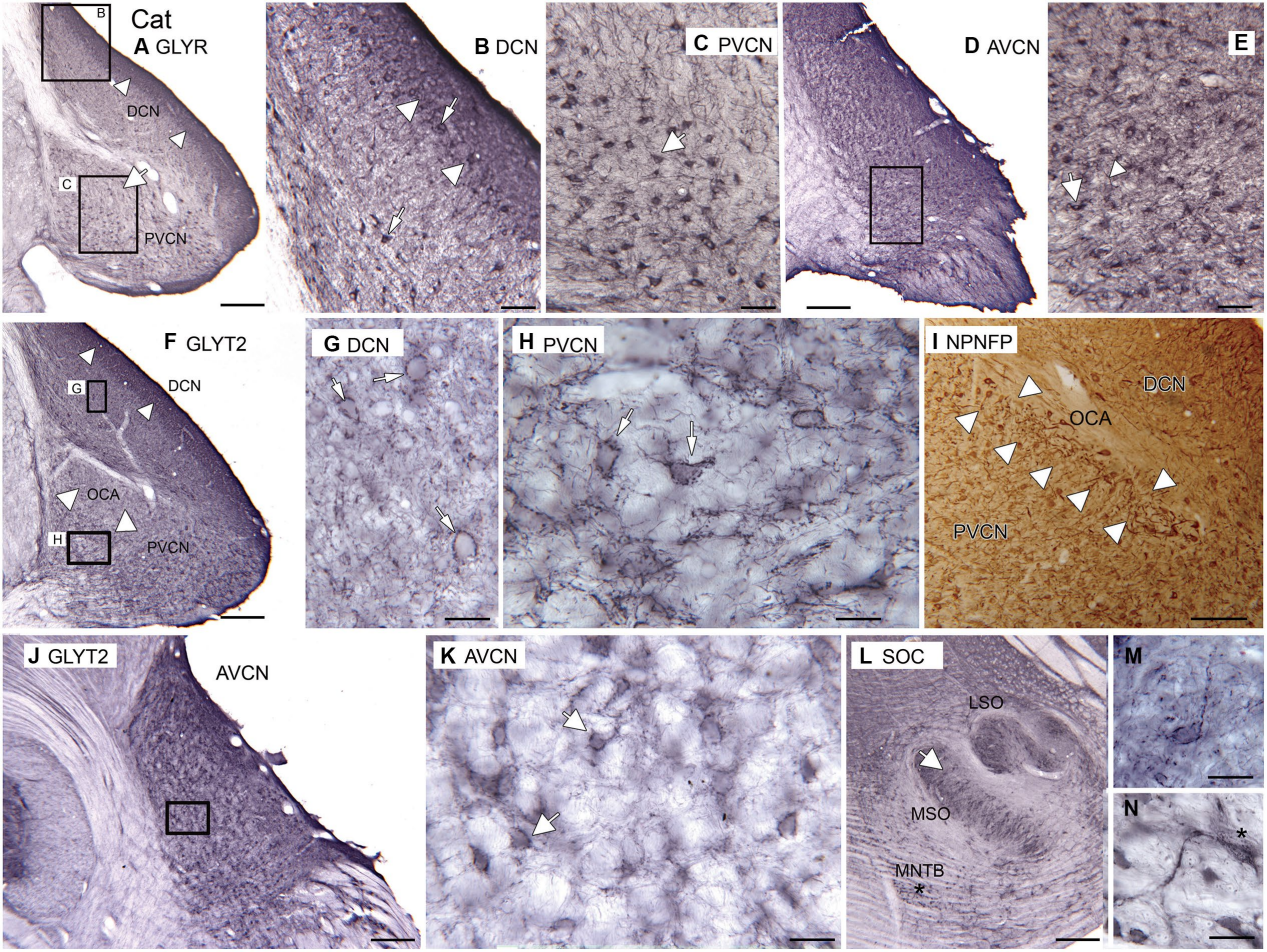
Glycine in the CN and SOC of the cat. **(A–E)** Immunoreactivity to GLYR, Abcam antibody. **(A)** Expression of glycine receptors in the DCN and PVCN. Note the dense band of staining at the outer border of the DCN (white arrowheads). There are also labeled somata in the PVCN. The white arrow shows a region with fewer GLYR-ir neurons. The rectangles show the locations of the higher magnification images of the DCN **(B)** and PVCN **(C)**. **(B)** DCN. The image shows a dense band of label at the outer border of the DCN (arrowhead) that includes immunolabeled somata; there are also many immunolabeled somata deep to that band. **(C)** PVCN. There are scattered immunolabeled somata of varying shapes (example at arrow); proximal processes are also immunolabeled. **(D)** Immunoreactivity to the glycine receptor in the AVCN. The rectangle shows the location of the higher magnification image in **E**. **(E)** There are immunolabeled somata (example at arrow) and processes (example at arrowhead) scattered throughout **E**. **(F–H,J–M)** Immunoreactivity to the GLYT2, Santa Cruz antibody. **(F)** Immunolabel in the DCN and PVCN. The arrowheads in the DCN show the darker band of label. In the PVCN, the white arrowheads show the location of the OCA, whose label is much sparser. The rectangles show the location of the images of DCN and ventromedial PVCN in **G,H**. **(G)** The immunolabeling outlines neurons in the DCN; the arrow indicates one example. **(H)** Neurons in the PVCN are outlined by immunolabel (examples at arrows). **(I)** NPNFP immunoreactivity in DCN and PVCN at the level of the OCA. The white arrowheads show the region with immunolabeled octopus cells. **(J)** GLYT2 immunolabel in the AVCN. The rectangle shows the location of the image in **K**. **(K)** Scattered neurons outlined by immunolabel; white arrows show two examples. **(L)** Immunoreactivity to GLYT2 in the SOC. The arrow indicates the location of the image in **M**; the asterisk is an alignment point for the image in **N** that shows immunoreactive neurons in the MNTB; there is a label in the cytoplasm as well as outlining the cells. Scale bars: **A,D,F,J,L** = 500 μm; **I** = 250 mm; **B,C,E** = 100 μm; **G,H,M,N** = 50 μm.

As expected, immunolabels for the glycine receptors and the neuronal glycine transporter suggest that there is glycinergic input to DCN, AVCN, and PVCN in cats. Figure 6A shows immunolabels for the glycine receptor in the DCN and PVCN. In the DCN, there is a very dark band of label about 250 μm wide extending to the surface of the brainstem (Figures 6A,B, white arrowheads). Below that band are scattered immunolabeled somata (Figure 6B, examples at arrows). In the PVCN, immunolabeled somata are sparser dorsomedially (large white arrow in Figure 6A, possible location of OCA) and denser ventrally. The immunolabeled somata are of a variety of shapes (example at arrow in Figure 6C). Proximal processes are also labeled, suggesting glycinergic input to both dendrites and somata. More rostrally, in the AVCN (Figures 6D,E), there are many labeled neurons throughout. The immunolabel outlines somata (Figure 6E, example at arrow); there are also immunostained fibers (arrowhead).

The pattern of immunolabeling with GLYT2 (Figures 6F–N) is also consistent with glycinergic input to the DCN (Figures 6F,G), PVCN (Figures 6F,H), and AVCN (Figures 6J,K). In the DCN, there is a band of darker label along the outer margin (Figure 6F, arrowheads), similar to the pattern seen with the GLYR antibody. Deeper in the DCN are immunolabeled puncta and somata outlined by immunoreactivity (Figure 6G, example at arrow). There is a region of PVCN with a sparse label (Figure 6F, white arrowheads); this region may correspond to the OCA. Outside of that region, there are outlined somata and puncta (Figure 6H, examples at arrows). Figure 6I shows a section immunolabeled for NPNFP; there is a good label of somata and dendrites of octopus cells. The OCA is indicated by white arrowheads. In the AVCN, immunolabeled somata are distributed throughout (Figures 6J,K). As in PVCN, there are neurons outlined by immunolabeling (Figure 6K, examples at arrows). To compare our data on the cat with earlier reports, we examined immunoreactivity to GLYT2 in the cat SOC. We found immunolabels in the LSO, MSO, and MNTB (Figure 6L). Figure 6M shows an example of a neuron in the MSO outlined by immunolabel. Figure 6N shows a very darkly immunolabeled neuron in the MNTB. This label may reflect cytoplasmic staining, similar to what was seen in glycinergic neurons in rats (Friauf et al., 1999).

## 4 Discussion

### 4.1 Overview

Our data suggest that, despite differences in DCN organization, glycine is an inhibitory transmitter in the CN of humans and chimpanzees. We saw widespread expression of glycine receptors on somata and processes of neurons in the DCN, PVCN, and AVCN, with a parallel distribution of glycinergic fibers and terminals. Our results in the cat are consistent with earlier anatomical and neurochemical studies of the CN in that species and also consistent with the differences in laminar organization of the DCN between cats and primates.

### 4.2 Humans and chimpanzees: DCN

In the human DCN, immunoreactivity to GLYRs showed a dense band of label that was composed of immunolabeled somata and processes. The location of the immunolabel was similar to antibodies to the neuronal glycine transporter. We found a similar band of immunolabel in the DCN with an antibody to NPNFP (Baizer et al., 2014; Figures 4A–C). In that study, we showed that the major neuronal components of this band are the fusiform cells and their processes. In humans, these somata and dendrites are found at all different orientations (Moore and Osen, 1979; Moore, 1980; Baizer et al., 2014), in contrast to the orderly arrangement of somata seen in other species. The similarity of the band of labels with GLYR, GLYT2, and NPNFP antibodies suggests that there are glycinergic inputs to the fusiform cells of the DCN. Such a result is consistent with what has been shown in other mammals (Caspary et al., 1987; Golding and Oertel, 1997; Ostapoff et al., 1997; Doucet et al., 1999; Caspary et al., 2005; Wang et al., 2011).

The pattern of immunolabel for GLYT2 in the DCN was similar in chimpanzees, with a band of label below the surface and parallel to it.

### 4.3 Humans and chimpanzees: PVCN, the OCA, and AVCN

We saw GLYR-immunolabeled neurons and processes in both the PVCN and AVCN. In the PVCN, there were labeled somata of several different shapes and sizes, likely including multipolar, bushy, and spindle-shaped neurons. The diversity of these neurons is similar to the diversity of Golgi-stained cells in the PVCN of the cat (see Figure 7 in Brawer et al., 1974) and mouse (see Figures 21, 22 in Webster and Trune, 1982).

In rats, two studies showed very little immunoreactivity to the neuronal glycine transporter in the OCA of rats (Zafra et al., 1995b; Friauf et al., 1999), suggesting that the OCA in that species lacks glycinergic input, a result consistent with electrophysiological data (Golding et al., 1995). However, Piechotta et al. (2001) showed glycine receptors on neurons around the periphery of the OCA; this may reflect an intermingling of octopus cells and other neurons outside of the OCA center. A lack of glycinergic input to octopus cells may not be true in all species; Wenthold et al. (1988) found expression of glycine receptors in octopus cells in the guinea pig. Octopus cells have been described in caudal PVCN in humans and chimpanzees (Heiman-Patterson and Strominger, 1985; Adams, 1986; Kulesza Jr, 2014). Several studies have found that octopus cells express the calcium-binding protein calretinin (CR; Adams, 1997; Bazwinsky et al., 2008; Felix et al., 2017), and our data from cats suggested that they also express NPNFP. We used those markers to try to locate octopus cells and the OCA in humans and chimpanzees. The borders of the OCA cannot be determined on the basis of immunoreactivity to CR or NPNFP since both are expressed in multiple neuron types (see Figure 6I, also Kulesza Jr, 2014; Baizer et al., 2018a). Comparison of the distributions of GLYR-ir and CR-ir neurons in humans and of NPNFP-ir neurons with GLYT2 immunoreactivity in chimpanzees does suggest that there is little or no glycinergic input to at least the central part of the OCA in both species.

We also saw immunoreactivity with glycine receptor antibodies on round somata, presumably spherical bushy cells, in the rostral AVCN. Immunoreactivity to glycine receptors has been seen on the somata of AVCN bushy cells in multiple species, including mice, guinea pigs, and macaque monkeys (Wenthold et al., 1988; Gomez-Nieto and Rubio, 2011; Lin and Xie, 2019).

### 4.4 CN and SOC in the cat: comparison to humans

Unlike humans, cats have a laminar DCN with an orderly arrangement of fusiform neurons (Brawer et al., 1974). There was a major difference in the pattern of immunolabeling in the DCN between cats and humans. In cats with both antibodies, there was an outer band of darker immunoreactivity extending to the surface. This is the layer in which the dendrites of the fusiform cells are found, and the label may reflect glycinergic input to the dendrites of fusiform cells. The pattern was different from the pattern in humans, in which there was a band of immunoreactivity below the surface, consistent with the differences in laminar organization of the DCN and the orientation of the fusiform cells between the two species.

Immunolabeling with both antibodies showed glycinergic input to PVCN and AVCN in cats. The presence of glycine receptors in the CN of cats was in agreement with Glendenning and Baker (1988). Glycinergic input to VCN bushy cells has also been reported in rhesus monkeys (Gomez-Nieto and Rubio, 2011). There also seemed to be little or no glycinergic input to octopus cells of the OCA, a result consistent with findings in the rat (Zafra et al., 1995b; Friauf et al., 1999). The immunolabeling pattern in the SOC we observed with the GLYT2 antibody was quite similar to the pattern of immunostaining seen in cats using an antibody to glycine (Spirou and Berrebi, 1997, cat, Figure 4). There were labeled neurons in the MNTB, as well as evidence for glycinergic input to neurons in the MSO.

### 4.5 What is the source of glycinergic input to the primate CN?

There is clear evidence for glycinergic input to the human CN; however, determining the source of that input is difficult in human tissue. Many studies have used antibodies to glycine itself to visualize glycinergic neurons (Aoki et al., 1988, rat; Gates et al., 1996, rat; Spirou and Berrebi, 1997, cat; Doucet et al., 1999, rat). However, such antibodies recognize glycine conjugated to glutaraldehyde and depend on having perfused animals with glutaraldehyde in the perfusate, which is not possible for the formalin-fixed tissue we used in our studies.

We must therefore rely on data from other species to consider possible sources of glycinergic input. There are glycinergic neurons in the DCN of other species; however, the existence of two classes of glycinergic neurons, tuberculoventral cells, and cartwheel cells, in human DCN has been challenged (Adams, 1986). In the VCN, commissural neurons and two populations of stellate cells are glycinergic (the D-stellate cells and the L-stellate cells; Wenthold, 1987; Ngodup et al., 2020). While multipolar/stellate cells have been identified in humans (Adams, 1986), available techniques have not allowed identification of the three classes of stellate cells (D-stellate, L-stellate, and M-stellate) or of their transmitters. In baboons, Moore et al. (1996) found many glycinergic neurons, presumably stellate cells, in the DCN and fewer in the VCN; such may exist in chimpanzees and humans. Another possible source of input to the CN is from the glycinergic neurons of the SOC. Glycinergic neurons have been found in several SOC nuclei: the MNTB, the LNTB, and the LSO (Aoki et al., 1988; Spirou and Berrebi, 1997). Neurons in various SOC nuclei project to the CN (Winter et al., 1989; Schofield, 1991, 1994); some of these neurons may be glycinergic. For both humans and chimpanzees, then, there is strong evidence of glycinergic input to the CN, but whether the glycinergic neurons are in the CN or SOC is not known.

## Data availability statement

The raw data supporting the conclusions of this article will be made available by the authors, without undue reservation.

## Ethics statement

The studies involving humans were approved by the Hamilton Integrated Research Ethics Board. The studies were conducted in accordance with the local legislation and institutional requirements. The participants provided their written informed consent to participate in this study. The animal study was approved by the Northwestern University Institutional Animal Care and Use Committee. The study was conducted in accordance with the local legislation and institutional requirements.

## Author contributions

JoB: Conceptualization, Investigation, Methodology, Resources, Supervision, Writing – original draft, Writing – review & editing. CS: Resources, Writing – review & editing. PH: Resources, Writing – review & editing. JaB: Resources, Writing – review & editing. SW: Resources, Writing – review & editing.

## Abbreviations

**Table:** 

AO	American optical
AR	Antigen retrieval
ACVN	Anterior ventral cochlear nucleus
CB	Calbindin
CN	Cochlear nuclei
CR	Calretinin
D	Dorsal
DCN	Dorsal cochlear nucleus
GLYR	Glycine receptor
GLYT2	Glycine neuronal transporter
Gr	Granule cell domain
-ir	Immunoreactivity
L	Lateral
LSO	Lateral superior olive
M	Medial
MNTB	Medial nucleus of the trapezoid body
MSO	Medial superior olive
NPNFP	Non-phosphorylated neurofilament protein
OCA	Octopus cell area (of the PVCN)
PBS	Phosphate-buffered saline
PMI	Post-mortem interval
PVCN	Posterior ventral cochlear nucleus
SOC	Superior olivary complex
SPON	Superior paraolivary nucleus
VCN	Ventral cochlear nucleus
